# MicroRNA-214 regulates smooth muscle cell differentiation from stem cells by targeting RNA-binding protein QKI

**DOI:** 10.18632/oncotarget.15189

**Published:** 2017-02-08

**Authors:** Yutao Wu, Zhoubin Li, Mei Yang, Bing Dai, Feng Hu, Feng Yang, Jianhua Zhu, Ting Chen, Li Zhang

**Affiliations:** ^1^ Department of Cardiology, First Affiliated Hospital, School of Medicine, Zhejiang University, Hangzhou, PR China; ^2^ Department of Lung Transplantation, First Affiliated Hospital, School of Medicine, Zhejiang University, Hangzhou, PR China

**Keywords:** miR-214, Quaking (QKI), stem cells, smooth muscle cells, cell differentiation

## Abstract

MicroRNA-214(miR-214) has been recently reported to regulate angiogenesis and embryonic stem cells (ESCs) differentiation. However, very little is known about its functional role in vascular smooth muscle cells (VSMCs) differentiation from ESCs. In the present study, we assessed the hypothesis that miR-214 and its target genes play an important role in VSMCs differentiation. Murine ESCs were seeded on collagen-coated flasks and cultured in differentiation medium for 2 to 8 days to allow VSMCs differentiation. miR-214 was significantly upregulated during VSMCs differentiation. miR-214 overexpression and knockdown in differentiating ESCs significantly promoted and inhibited VSMCs -specific genes expression, respectively. Importantly, miR-214 overexpression in ESCs promoted VSMCs differentiation *in vivo*. Quaking (QKI) was predicted as one of the major targets of miR-214, which was negatively regulated by miR-214. Luciferase assay showed miR-214 substantially inhibited wild type, but not the mutant version of QKI-3-UTR-luciferase activity in differentiating ESCs, further confirming a negative regulation role of miR-214 in QKI gene expression. Mechanistically, our data showed that miR-214 regulated VSMCs gene expression during VSMCs differentiation from ESCs through suppression of QKI. We further demonstrated that QKI down-regulated the expression of SRF, MEF2C and Myocd through transcriptional repression and direct binding to promoters of the SRF, MEF2c and Myocd genes. Taken together, we have uncovered a central role of miR-214 in ESC-VSMC differentiation, and successfully identified QKI as a functional modulating target in miR-214 mediated VSMCs differentiation.

## INTRODUCTION

Regenerative medicine is an interdisciplinary field with the ultimate goal to repair, replace, or regenerate cells, tissues and organs that are lost or damaged due to disease, injury, or ageing. Vascular tissue engineering has the potential to provide biological substitutes for repairing or replacing the damaged or blocked vessels in patients [1–4]. However, the lack of autologous vessels for complicated surgeries remains problematic. Embryonic stem cells (ESCs), one of the most promising stem cell sources, are pluripotent derivatives of the inner cell mass of blastocysts. They have the capacity for unlimited growth and self-renewal and the ability to differentiate into a wide range of specialized cell types including vascular endothelial cells [5, 6] and smooth muscle cells (SMCs) [7–10].

Vascular smooth muscle cells (VSMCs) are involved in the physiological and pathological processes of the vascular system. Differentiated VMSCs demonstrate a very low rate of proliferation, appropriate contractility to contractile cues, and express SMC-specific genes, such as smooth muscle α-actin (SMA), smooth muscle myosin heavy chain (SMMHC), 22-kDa SMC lineage-restricted protein (SM22), and calponin. To investigate VSMCs differentiation closely, we have established an excellent and simple *in vitro* VSMCs differentiation model from ESCs by using collagen as cellular base membrane and demonstrated that differentiation of mouse ESCs towards VSMCs lineage is mediated by a fairly complicated regulatory circuitry composing of transcription factors (Sp1 [11]) and DNA/RNA binding proteins (heterogeneous-nuclear ribonucleoprotein and A1 [12]). Although these findings have significantly improved our understanding regarding VSMCs differentiation and cardiovascular system development, the detailed molecular mechanisms of VSMCs differentiation from ESCs have not been fully clarified.

MicroRNAs (miRs), an emerging class of highly conserved, noncoding small RNAs, which regulate gene expression at the post-transcriptional level by inhibiting the translation of protein from mRNA or by promoting the degradation of mRNA [13]. miRs are involved in a variety of basic biological processes such as cardiogenesis, hematopoietic lineage differentiation, and oncogenesis [14–17]. Recent studies have indicated that many miRs are highly expressed in vascular system and involved in the VSMCs proliferation and differentiation [18, 19]. Several reports demonstrated that miR-143 and miR-145 were enriched in VSMCs and played a significant role in regulating the phenotypic switching of VSMCs [20]. miR-221 and miR-222 were also implicated in modulation of VSMCs differentiation. In cultured VSMCs, miR-221 and miR-222 expression can be transcriptionally induced by PDGF, and both miRNAs play a role in VSMCs phenotypic switch [21]. miR-34a and miR-22 were also reported to regulate VSMCs differentiation from stem cells by targeting sirtuin 1 and Methyl CpG–Binding Protein, respectively [22, 23]. However, the significance and exact role of individual miRNA in VSMCs differentiation remains to be further elucidated.

In the present study, we demonstrated for the first time that miR-214 plays an important role in murine ESCs differentiation towards VSMCs *in vitro* and *in vivo*. Meanwhile, our data also revealed that QKI was regulated by miR-214 in VSMCs differentiation.

## RESULTS

### Important role of miR-214 in VSMCs differentiation from ESCs *in vitro*

**Figure 1:**
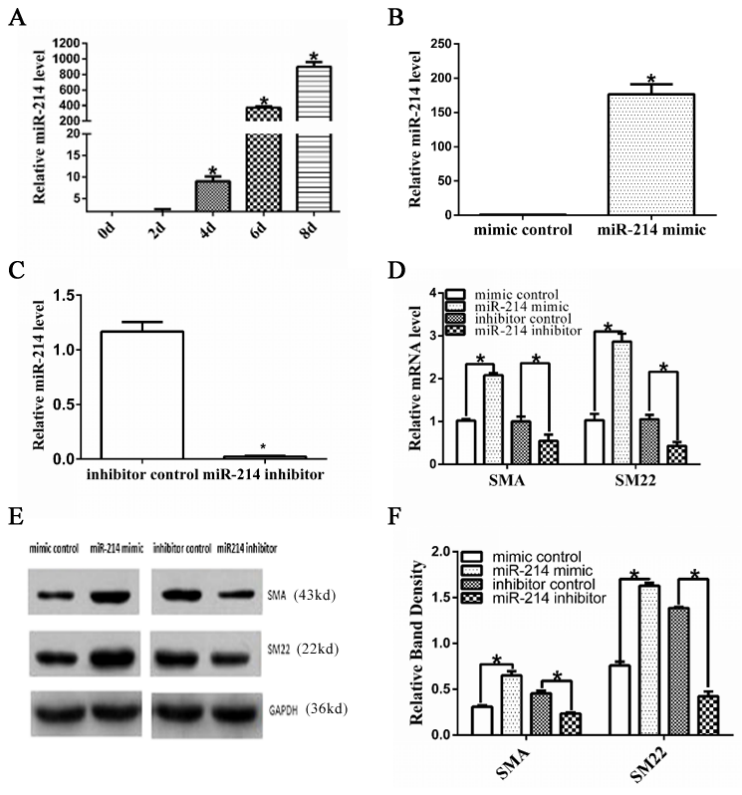
Important role of miR-214 in VSMCs differentiation from ESCs **A**. Up-regulated miR-214 during VSMCs differentiation from ESCs. Mouse ESCs were seeded on Collagen IV coated dishes and cultured in differentiated media, the expression of miR-214 during VSMCs differentiation was assessed through RT-qPCR analyses. (Data are means ± SEM. (n=3), *p<0.05. **B-F**. SMC-specific gene expression was modulated by miR-214. ESCs were induced to differentiate towards VSMCs for 4 days, and the differentiating ESCs were transfected with either miRNA mimics negative control (mimic control in short), miR-214 mimics, or miRNA inhibitor negative control (inhibitor control in short) or miR-214 inhibitor, respectively. Transfected cells were cultured in VSMCs differentiation medium for another 48 hours. Total RNAs or proteins were harvested and subjected to RT-qPCR (B-D) or Western Blotting (E-F) analyses, respectively. The data presented here are representative (E) or mean±S.E.M. of three to six independent experiments. *P<0.05.

The expression level of miR-214 was upregulated during VSMCs differentiation from ESCs (Figure 1A). To elucidate the potential function of miR-214 during ESC-SMC differentiation, the chemically synthesized miR-214 inhibitor or miR-214 mimic was used in the miR-214 loss-of-function and gain-of-function experiments, respectively. As expected, miR-214 mimics and miR-214 inhibitor transfection resulted in a significant increase and decrease of miR-214 expression in the differentiating ESCs (Figure 1B, 1C). As a result, miR-214 overexpression dramatically increased the expression of VSMCs markers such as SMA and SM22 at both mRNA (Figure 1D (quantification)) and protein levels (Figure 1E, 1F), whereas, miR-214 inhibition significantly downregulated the expression of VSMCs markers (Figure 1D-1F). These data suggest an important role of miR-214 in VSMCs differentiation from ESCs.

### miR-214 is involved in VSMCs differentiation *in vivo*

**Figure 2:**
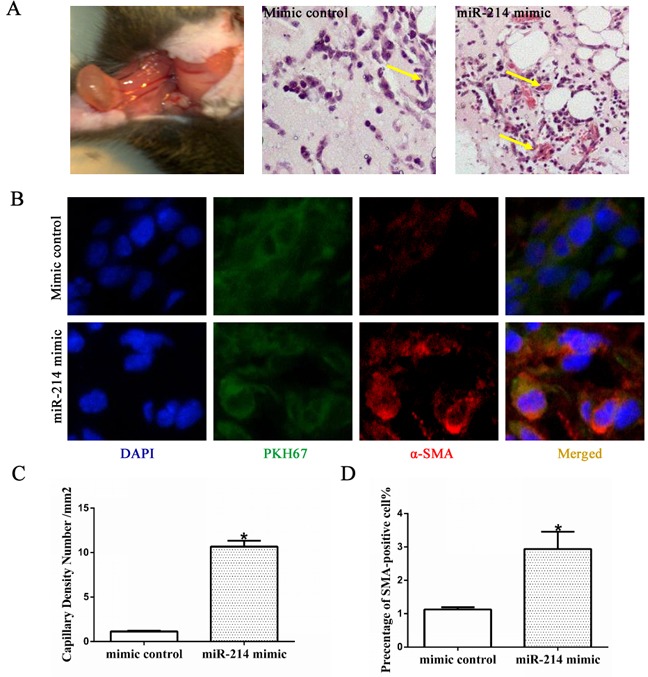
miR-214 promotes VSMCs differentiation *in vivo* ESCs were seeded on collagen IVcoated plates and cultured in DM media for 4 days. After then the miR-214 mimic or mimic control were introduced to the cells by transfection. Seven days later, the cells were labeled with PKH67, followed by *in vivo* Matrigel implantation. **A**. Matrigel plug and H&E staining. Far left, the Matrigel plug when harvested at 13 days post-implantation; H&E images of the Matrigel plugs with cells transfected with control mimics (middle) or miR-214 mimics (far right), respectively. More vasculture-like structures were observed in the Matrigel implants of miR-214 mimics-transfected cells **C**. **B** and **D**. More SMA-positive cells were observed in the Matrigel plugs implanted with miR-214 over-expressing ESCs. Frozen section of Matrigel implants were subjected to immuno-fluorescence staining using antibody against SMA. DAPI was used to stain the cell nucleus. Representative images (B) and quantitative data (D) of the percentage of SMA-positive cells were presented here, respectively. Note: cells with green fluorescence signal indicate PKH67-labelled cells (implanted cells) within Matrigel plugs. The percentage of PKH67-labelled SMA-positive cells per field were examined by two well-trained independent investigators blinded to the treatments, from four random high power fields (200x) in each section, three sections from each implant and four implants for each group, *p<0.05.

To further explore the functional importance of miR-214 in VSMCs differentiation *in vivo*, ESCs labelled with PKH67 were transfected with mimic controls or miR-214 mimic and subcutaneously injected into C57BL/6J mice as described in our previous study [12, 25]. Some vascular structures were observed with miR-214 overexpression, while there were no defined vascular structures in control group (Figure 2A and the quantification in Figures 2C). Immunofluorescence staining with antibody of SMA showed that compared with control, more SMA-positive cells were presented in the Matrigel implants mixed with miR-214 overexpressing ESCs (Figure 2B and 2D). As expected, the majority of cells in the Matrigel implants were PKH67-positive (green signal), indicating its exogenous origins (Figure 2B). Taken together, these data clearly suggested a central role of miR-214 in VSMCs differentiation from stem cells *in vivo*.

### Target gene, QKI, is suppressed by miR-214

**Figure 3:**
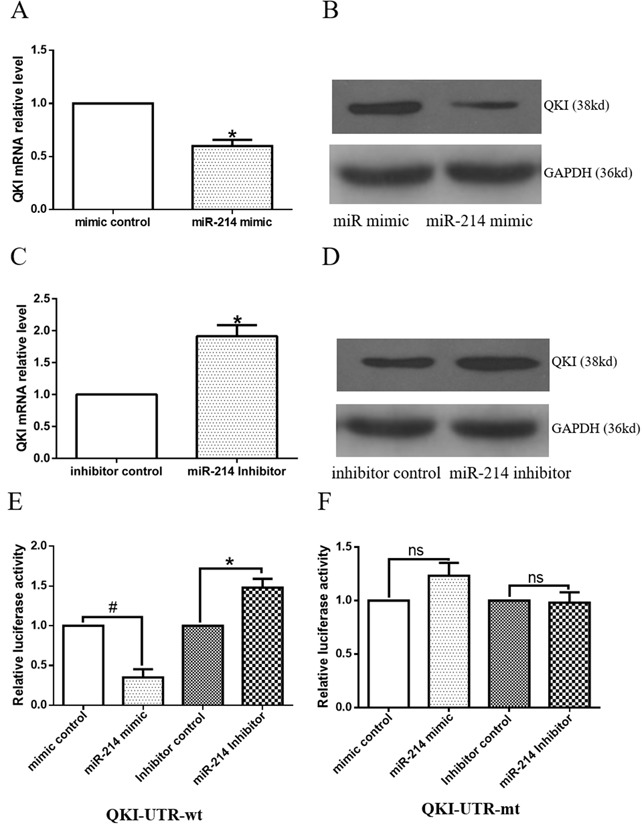
Target gene, QKI, is negatively regulated by miR-214 **A-D**. Modulations of miR-214 expression levels negatively regulate QKI gene expression. Day 2~3 differentiating ESCs were transfected with miR-214 mimic or inhibitor, or respective negative controls, and cultured in VSMCs differentiation medium for 48 to 72 hours. Total RNA and protein were harvested and subjected to RT-qPCR (A and C) and Western blot (B and D) analyses, respectively. (Data are means ± SEM. (n=3), *p<0.05). **E-F**. ESCs were forced to differentiate towards VSMCs for 4 days, and co-transfections of miR-214 mimic with the luciferase plasmid of the QKI-UTR-wt or QKI-UTR-mt were performed. Forty-eight hours later, cells were harvested and subjected to luciferase analyses, (data are means ± SEM. (n=3), *p<0.05, # p<0.01).

To further investigate the underlying mechanisms of miR-214-mediated VSMCs differentiation, the potential mRNA targets of miR-214 were scrutinized. QKI was emerged as one of the top targets of miR-214 in several computational algorithmic databases, such as Targetscan (www.targetscan.org), pictar (http://www.pictar.org), and miRanda (www.microrna.org), and two highly conserved binding sites for miR-214 have been identified within the 3′UTR of QKI-6/7, but not QKI-5. Notably, both miR-214 binding sites are localized within the shared 3′UTR region of QKI-6 and QKI-7. Indeed, QKI gene and protein expression levels were significantly down-regulated or up-regulated by over-expression or inhibition of miR-214 in the differentiating ESCs (Figure 3A, 3B, 3C and 3D), respectively, demonstrating a negative regulatory role of miR-214 in QKI gene expression. To investigate direct interaction between miR-214 and QKI, the 3′UTR of QKI contained the binding sites of miR-214 was cloned into a luciferase reporter. Data from our miRNA reporter assay showed that the activity of luciferase from construct harbouring the QKI 3′UTR was significantly down-regulated or -up-regulated by miR-214 over-expression or inhibition, respectively (Figure 3E) while the luciferase activity of reporters with QKI 3′UTR mutation was little change (Figure 3F). Altogether, above data has firmly confirmed that QKI is a direct target of miR-214, which was inhibited by miR-214 during VSMCs differentiation.

### QKI repression is required for miR-214 induced VSMCs gene expressions

**Figure 4:**
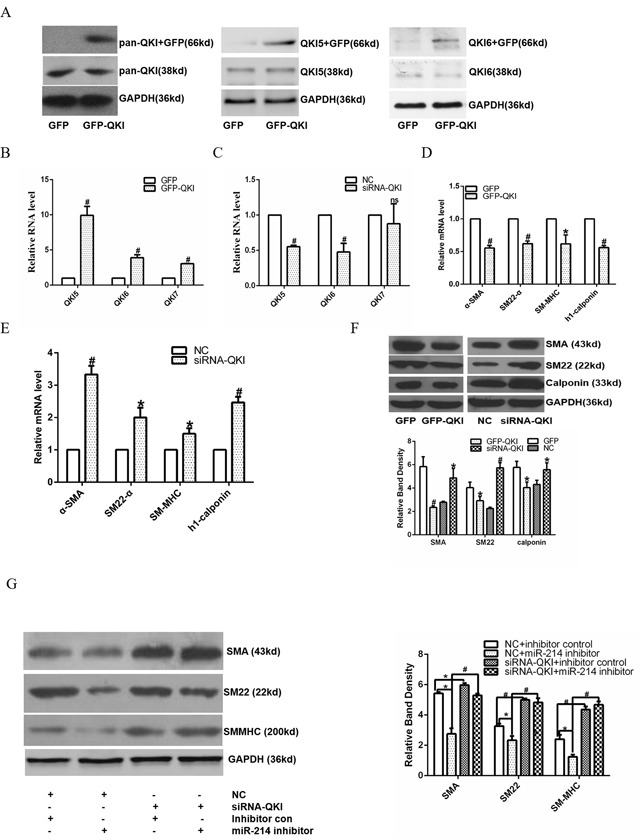
miR-214 mediated VSMCs gene expression through modulating QKI **A-F**. Over-expression of QKI up-regulates VSMCs gene expression, while knockdown of QKI down-regulates their expression. Day 2~3 differentiating ESCs were transfected with control (GFP) or QKI over-expression plasmid (GFP-QKI), or control siRNA (NC) or QKI specific siRNA (siRNA-QKI), respectively. Transfected cells were cultured in VSMCs differentiation medium for another 48 or 72 hours. Total RNAs and proteins were harvested and subjected to RT-qPCR and Western Blotting analyses, respectively. QKI and its isoforms were upregulated (A and B), resulting in VSMCs marker genes downregulation (D and F), while siRNA-QKI (C) increased marker genes expression (E and F) at both RNA and protein level. (G) Knock-down of QKI abolished the inhibitory effect of miR-214 inhibition on VSMCs gene expression. Day 2~3 differentiating ESCs were co-transfected with miR-214 inhibitor, QKI siRNA or respective negative control as indicated in the figure, and cultured for further 48-72 hours. Total proteins were harvested and subjected to Western blotting assays. The data presented here are representative or mean±S.E.M. of three independent experiments, *P<0.05, **#**P<0.05 (versus respective control)

We have demonstrated that QKI is the mRNA target of miR-214 during VSMCs differentiation. To investigate the potential role of QKI in VSMCs differentiation, QKI over-expression in the differen-tiating ESCs was conducted by using QKI over-expression plasmid (pGFP-QKI). The QKI proteins belong to the heteronuclear ribonucleoprotein particle K (hnRNPK) homology (KH) domain family of RNA binding proteins. Three major alternatively spliced mRNAs (5, 6, and 7 kb) encoding QKI-5, QKI-6, and QKI-7 that differ in their C-terminal 30 amino acids and 3′UTR are transcribed from QKI gene. Data showed that QKI over-expression significantly upregulated QKI and its isforms (QKI5 and 6, Figure 4A and 4B), resulting in down-regulation of VSMCs specific marker expressions (Figure 4D and 4F), while QKI inhibition (Figure 4C) by siRNA dramatically up-regulated them (Figure 4E and 4F), both at mRNA and protein level. Moreover, data from the co-transfection experiments showed that while QKI knockdown or miR-214 inhibition alone in the differentiating ESCs significantly up-regulated or down-regulated various VSMCs specific gene expression, respectively, inhibition of QKI almost abolished the inhibitory effects of miR-214 inhibition on VSMCs specific gene expression (Figure 4G), suggesting that miR-214 regulates VSMCs gene expression through modulation of QKI.

### VSMCs transcription factors are suppressed by QKI

**Figure 5:**
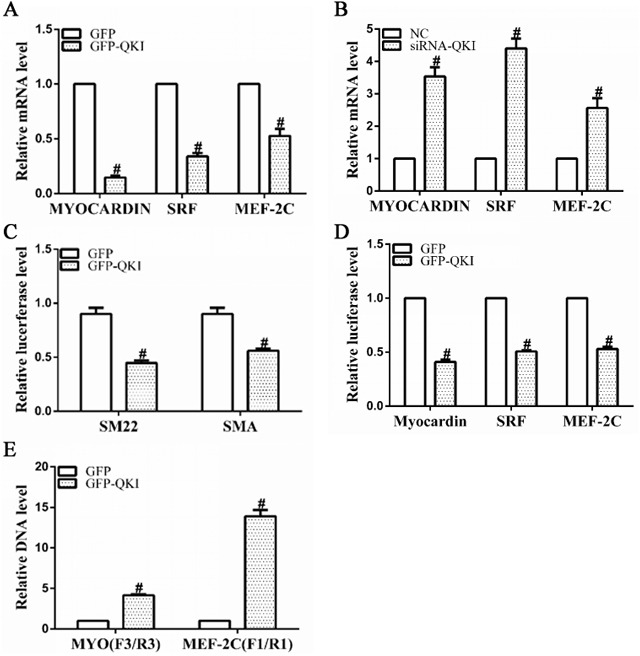
VSMCs transcription factor gene expressions were negatively and transcriptionally regulated by QKI **A, B**. mRNA levels of SRF, MEF2c and myocardin were significantly down-regulated by QKI over-expression, but up-regulated by QKI suppression. Total RNAs were harvested as described in Figure 4A and 4B. **C, D**. QKI regulated the gene promoter activities of VSMCs markers and transcription factors. Day 2~3 differentiating ESCs were transfected with luciferase reporter plasmids pGL3-SMA-Luc, pGL3-SM22-Luc, pGL3-SRF-Luc, pGL3-MEF2c-Luc or pGL3-Myocd-Luc (0.15μg/2.5×10^4^ cells) together with GFP-QKI or GFP (0.2μg/2.5×10^4^ cells). Luciferase activity assays were detected 48 hours after transfection. The data presented here are mean±S.E.M. of three to six independent experiments. *P<0.05 (vs. control). **E**. QKI binds directly to the promoter regions of MEF2c and myocardin genes. ChIP assays were performed using antibodies against QKI or normal rabbit IgG, respectively, as described in the Methods section. PCR amplifications of the specific promoter regions of MEF2c or Myocd gene. The data presented here are mean±S.E.M. of four independent experiments. *P<0.05, #P<0.05 (vs. control).

SRF, MEF2c and myocardin (Myocd) are well-known transcription factors to activate VSMCs gene expression during cardiovascular system development. Data shown in Figure 5A, 5B revealed that gene expression levels of all three factors were significantly decreased by QKI overexpression, but increased by QKI knockdown, suggesting QKI may have a direct role in regulation of these transcription factors during VSMCs differentiation. To confirm such possibility, luciferase activity assays by using SMA, SM22, SRF, MEF2c and Myocd gene reporter plasmids (pGL3 -Luc-SMA, pGL3 -Luc-SM22, pGL3 -Luc-SRF, pGL3 -Luc-MEF2c and pGL3 -Luc-Myocd) generated in our previous study [12] were conducted in the differentiating ESCs. Data showed that the QKI over-expression significantly decreased SMA, SM22, SRF, MEF2c or Myocd gene promoter activities (Figure 5C, 5D), indicating that QKI may inhibit transcriptional activity of these genes. To further investigate if QKI can directly bind to the promoters of SRF, MEF2c and Myocd, and its potential binding region(s) of QKI within these three gene promoters, CHIP assays with QKI antibody were conducted and two pairs of primers for each gene were used to amplify target DNA. A set of specific primers (4 pairs) spanning through the respective promoter region of SRF, MEF2c and Myocd as described in our previous study [12] were used in our preliminary study and the best primer pairs were chosen to use in the following experiments. Data shown in Figure 5E revealed that QKI directly binds to the promoter regions between -1335 and -1263 of MEF2c gene or -708 and -620 of Myocd gene, respectively. Taken together, these findings strongly imply that QKI transcriptionally suppress VSMCs transcription factor gene expression during VSMCs differentiation from stem cells.

## DISCUSSION

**Figure 6:**
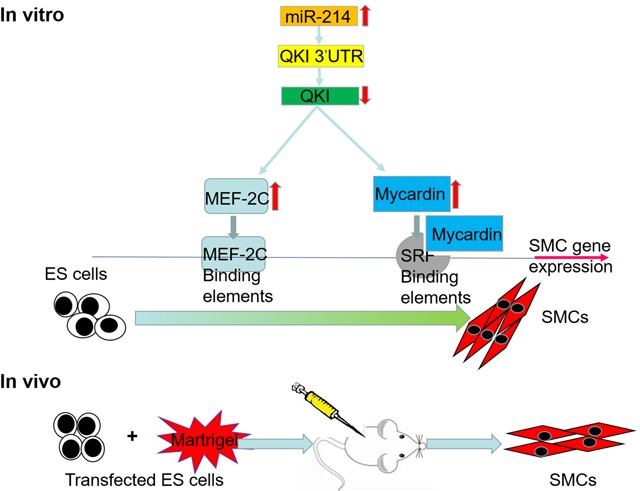
Schematic representation of the mechanism by which miR-214 regulates ESCs differentiation towards VSMCs by targeting QKI

It has been shown that ESCs can differentiate into VSMCs via mechanisms controlled by different signal transduction pathways [7–10]. Despite enormous efforts have been put into this field in the past decade, our understandings into the molecular mechanisms undergoing VSMCs differentiation are still far from complete. In the present study, we have advanced our knowledge of the molecular mechanism mediating VSMCs differentiation by confirming an important role of miR-214 in promoting VSMCs specific gene expression and VSMCs differentiation from murine ESCs *in vitro* and *in vivo*. Meanwhile, we have identified QKI as the mRNA target of miR-214, further demonstrated that miR-214 inhibited its target gene QKI, during ESC-SMC differentiation. Mechanistically, we have clearly defined that QKI is a potential transcription repressor of SMC-specific gene regulation. Moreover, our data also revealed that QKI inhibit VSMCs transcription factors (SRF, MEF2c and Myocd) at transcriptional level. Taken together, our data provided evidence to support that miR-214 promotes VSMCs differentiation by suppressing QKI (Figure 6).

### miR-214 and SMC differentiation

miR-214 was found to regulate the polycomb protein Ezh2 in skeletal muscle and embryonic stem cell [26], and suppress stem-like traits in human hepatocellular carcinoma though targeting beta-catenin pathway [27]. Importantly, it has been recently reported that miR-214 was specifically expressed during neuroblastoma differentiation, cortical development and embryonic stem cells differentiation [24, 28], and that miR-214 suppressed osteogenic differentiation [29]. All the aforementioned studies indicated that miR-214 was one of major regulators during stem cells differentiation. However, up to now, the mechanism roles of miR-214 in ESC-SMC differentiation are still not well understood. By using our well-established VSMCs differentiation system and miR array technique, miR-214 was emerged as a potential miR candidate to regulate VSMCs differentiation. By utilizing gain/lose-of function analyses, we confirm miR-214 promotes VSMCs differentiation from murine ESCs *in vitro*. Furthermore, we also provide clear evidence to support that miR-214 enhances embryonic VSMCs differentiation *in vivo* by using another well-established *in vivo* Martigel implantation model. These observations clearly imply that miR-214 played an important role in ESC-SMC differentiation.

### Target gene, QKI, is suppressed by miR-214 during VSMCs differentiation

It has been suggested that miR-214 may inhibit angiogenesis by targeting QKI and reduce angiogenic growth factor release [30]. Eric van et al proposed that QKI is a central regulator of VSMCs phenotypic plasticity and that intervention in QKI activity can ameliorate pathogenic, fibroproliferative responses to vascular injury. In our system, we have provided clear and solid evidence to suggest that QKI is a functional target gene of miR-214 during VSMCs differentiation from stem cells. Such notion has been supported by following observations: the expression of QKI gene and protein levels were negatively regulated by miR-214 in the differentiating ESCs, and the QKI 3′UTR reporter activity was reversely modulated by miR-214.

### QKI regulates VSMCs differentiation gene expression through a transcriptional mechanism

We provided clear evidence in this study to support that QKI inhibits VSMCs differentiation gene expression through a transcriptional mechanism. The RNA-binding protein Quaking (QKI) is a member of the highly conserved signal transduction and activator of RNA (STAR) family of RNA-binding proteins, which has been found to affect pre-mRNA splicing, mRNA turnover, and translation [31]. Previous study found that the production of over one-third of abundant circRNAs is dynamically regulated by the alternative splicing factor, QKI, which itself is regulated during EMT [31]. circRNAs which are formed by non-sequential back-splicing of pre-mRNA transcripts are a wide-spread form of non-coding RNA (similar as miRNA) in animal cells. Mutations in the mouse QKI locus can result in the embryonic lethality and QKI is a critical regulator of colon epithelial differentiation as well [32–34]. In the present study, we have provided clear evidence that QKI is an important VSMCs differentiation mediator, and that QKI regulates SMC-specific gene expression (SMA, SM22, SRF, MEF2c and myocardin) during VSMCs differentiation through a transcriptional repression manner.

Taken together, we have successful uncovered a novel regulatory role of miR-214 in VSMCs differentiation from ESCs *in vitro* and *in vivo*, and provided sufficient evidence to support our hypothesis that miR-214 regulates ESC-SMC differentiation though suppressing its target gene QKI. Moreover, we have revealed that QKI down-regulated VSMCs gene expression through a transcriptional mechanism. However, it is noteworthy to mention that although we have provided some direct evidence to show that QKI regulates SMC-specific transcription factor gene transcriptional activity through direct binding to the promoter regions of these genes (Myocd and MEF2c), the detailed essential binding elements of QKI or exactly QKI binding motifs within these gene promoter regions remains to be further identified. Nevertheless, our findings will significantly increase our understanding of the molecular mechanisms in VSMCs differentiation and benefit for future application in regenerative medicine.

## MATERIALS AND METHODS

### Materials

Antibody against QKI was purchased from Santa Cruz Biotech (sc-103851), USA. Antibodies against QKI5, QKI6, QKI7 were from Millipore (AB9904, AB9906, AB9908), USA. Antibody against Smooth Muscle Myosin Heavy Chain (SMMHC) was from AbD Serotec (Rabbit, AHP1117). Antibodies against SM22 (rabbit, Ab14106) and calponin (rabbit, Ab46794) were from Abcam, UK. Antibodies against GAPDH (mouse) and monoclonal anti-α smooth muscle actin (SMA) (Clone 1A4, A5228) were from Sigma. All secondary antibodies were from Santa Cruz Biotech.

### Cell culture and differentiation

Detailed protocols for mouse ESCs (ES-D3 cell line, CRL-1934; ATCC, Manassas, VA) culture and VSMCs differentiation were previously described [8, 24]. Briefly, ESCs were seeded on gelatin (Sigma) coated flasks and cultured in culture medium (CM) which included Dulbecco's Modified Essential Medium (DMEM) (ATCC), 10% Embryomax Foetal Bovine Serum (FBS) (Millipore), 10ng/ml Leukaemia Inhibitor Factor (LIF) (Millipore), 0.1mM 2-mercaptoethanol (2-ME) (Life Technologies™), 100U/ml penicillin and 100μg/ml streptomycin (Life technologies) and 2mM Glutamine (Life technologies). They were split every other day in a ratio of 1:10. For VSMCs differentiation, undifferentiated ESCs were seeded on mouse collagen IV (5μg/ml) coated flasks or plates in differentiation medium (DM) that contains α-minimal essential medium (aMEM Invitrogen) supplemented with 10% FBS, 0.05mM 2-ME, 100U/ml penicillin, 100μg/ml streptomycin and 2mM glutamine. DM was refreshed every day after the second day of differentiation. The cells were cultured in DM for 2-8 days after which they were harvested and further analyzed.

### Indirect immunofluorescent staining for cells

Cells were labelled with isotype IgG control or antibody against SMA, and visualized using appropriate secondary antibody conjugated with tetramethylrhodamine isomer R (TRITC, DAKO). Cells were counterstained with 4′, 6-diamidino-2-phenylindole (DAPI; Sigma) and mounted in Fluoromount-G (Cytomation; DAKO, Glostrup, Denmark). Images were examined using SP5 confocal microscope with Plan-NEOFLUAR 63x objective lenses and Leica TCS Sp5 software (Leica, Germany) at room temperature, and were processed with Photoshop software (Adobe).

### Immunoblotting

Cells were harvested and lysed in lysis buffer (50mM Tris-Cl pH 7.5, 150mM NaCl, 1 mM EDTA pH 8.0) supplemented with protease inhibitors and 0.5% Triton and sonicated to obtain whole cell lysate. Equal amount of proteins (40ug) was resolved by SDS-PAGE with 4%~20% Tris-Glycine gel (Invitrogen, Carlsbad, CA, USA) and subjected to standard Western blot analysis. The resulting blots were subjected to densitometric analysis with Image J software. To ensure equal protein loading, the GAPDH protein was used as internal control. Relative protein expression level was defined as the ratio of target protein expression level to GAPDH expression level with that of the control sample set as 1.

### Real time quantitative PCR (RT-qPCR) for mRNA and microRNAs

Real-time quantitative PCR (RT-qPCR) was conducted as previously described [5]. Briefly, total RNA containing small RNAs (microRNAs) was extracted from cells using mirVana™ Protein and RNA Isolation System™ Kit (Applied Biosystems, Ambion Inc) or TRI reagent (Sigma) according to the manufacturer's instructions. Total RNA and microRNA specific cDNA synthesis was performed with Prime Script RT Master Mix Perfect Real Time kit (DRR036A, Takara, China) and miRNA RT-PCR Kit (RR716, Takara, China), respectively. The resultant cDNA was diluted to a working concentration of 5ng/μl and stored at -20°C. The qRT-PCR reaction was run on ABI Prism 7500 system, using NCode™ EXPRESS SYBR® GreenER™ qPCR SuperMix Universal for miRNA RT-qPCR or Takara premix Ex Taq II (DRR820A, Takara, China) for others. Sequence for each primer was shown in Supplementary Table 1. Expression level of relative mRNA or microRNA was defined as the ratio of target gene expression level or microRNA expression level to 18S or U6 snRNA expression level, respectively, with that of the control sample set as 1.0.

### *In vivo* VSMCs differentiation and immunofluorescent staining assay

The procedures for *in vivo* VSMCs differentiation were similar to that as described in previous study [12, 22, 23, 25]. Briefly, control or miR-214 over-expression ESCs (10^6^ in 50μl aMEM) were labeled as green fluorescence PKH67 (sigma), mixed with 50μl of Matrigel (Becton Dickinson Labware) and PDGF-BB (100ng/ml) at 4°C, and subcutaneously injected into C57BL/6J mice. After 10~13 days, mice were euthanized and the implants (Matrigel plugs) were harvested and frozen in liquid nitrogen for future using. All animal experiments were performed according to protocols approved by the Institutional Committee for Use and Care of Laboratory Animals. For immunofluorescent staining, sections were cut at 8 μm for optimum cutting temperature compound-embedded Matrigel implants, every 40 μm along the longitudinal axis of Matrigel plugs, and numbered. Given numbered sections (for instance, sections 5, 15 and 25) were subjected to immunohistological analyses with respective antibody. Briefly, frozen sections were air-dried for at least 30 minutes, followed by fixed in cold acetone for 15 minutes. The sections were then rinsed in PBS and blocked with 5% BSA in PBS (Dako) for 1 hr at room temperature in a humid chamber. The incubation with primary antibodies (SMA) or IgG controls diluted in blocking buffer was performed in a cold room (4°C) overnight. Followed by incubation with TRITC conjugated secondary antibodies, sections were then incubated with DAPI (1:1000, Sigma) for 5 minutes. Images were assessed with Olympus IX71 imaging microscope and CellSens Dimension software (all Olympus Corporation.) at room temperature, and were processed with Photoshop software (Adobe). The percentage of PKH67-labelled SMA positive cells per field were counted by two well-trained independent investigators blinded to the treatments, from four random high power fields (200x) in each section, three sections from each implant and four implants for each group.

### microRNA and plasmids transfection

Either miRNAs inhibitors or precursors and miRNA negative controls transfections have been performed using Lipofectamine RNAiMAX (Invitrogen), at a final concentration of 30 nM, according to the manufacturer's protocol. Transfected cells were cultured on collagen IV coated flasks or plates for 48~72 hours in DM to allow VSMCs differentiation. All miRNA inhibitors or precursors and respective negative controls were purchased from Ambion. Control (GFP) and QKI overexpression (p-GFP-QKI, BAIAO shanghai China) plasmids were transfected into differentiating ESCs using Lipofectamin 3000 (Invitrogen) according to the manufacturer's instructions.

### Luciferase reporter assay

For the luciferase reporter assays, 3 × 10^4^ ES cells were seeded on collagen-coated well of a 12-well plate in DM. 72 h later, cells were transfected with the QKI 3′UTR Lenti-reporter-Luc Vector (ABM, QKI-UTR-wt) or QKI 3′UTR Lenti-reporter-Luc vector with mutated miRNA 214 binding site (QKI-UTR-mt), and the indicated miRNAs or controls. Briefly, 0.33 μg/well of the reporter plasmids were co-transfected with the miR-214 mimics, miR-214 inhibitor or respective controls (2 μl/well) using jetPRIME® (Polyplus-transfection SA) according to the protocol provided. pRenilla (0.1μg/well) was included in all transfection assay as internal control. Luciferase and Renilla (Promega) activity assays were detected 48hr after transfection using a standard protocol [7]. Relative luciferase unit (RLU) was defined as the ratio of luciferase activity to Renilla activity with that of control set as 1.0.

### Chromatin immunoprecipitation (ChIP) assays

The ChIP assays were performed as previously described [5, 6, 8, 9]. Briefly, differentiating ESCs transfected with control (pGFP) or QKI over-expression (pGFP -QKI) plasmids were treated with 1% (v/v) formaldehyde at room temperature for 10 min and then quenched with glycine at room temperature. The medium was removed, cells were harvested and sonicated. The sheared samples were diluted into 1 ml immunoprecipitation buffer, and immunoprecipitations were conducted with antibodies raised against QKI (ChIP grade, Abcam), together with single-strand salmon sperm DNA saturated with protein-G-Sepharose beads. Equal amount (2μg/immunoprecipitation) of normal rabbit IgG or mouse IgG was used as control. The immunoprecipitates were eluted from the beads using 100 μl elution buffer, and immunoprecipitaed DNA was extracted, purified, and then used to amplify target DNA sequences by RT-qPCR using specific primers (Supplementary Table 1). Relative DNA level (or promoter DNA enrichment) was defined as the ratio of immunoprecipitated promoter DNA level to its input level with that of the control sample (pGFP) set as 1.0. The data was obtained from four independent experiments.

### Statistical analysis

Data were expressed as mean±SEM and analyzed using a two-tailed student's *t*-test for two-group comparison or one-way ANOVA followed by Tukey's HSD multiple comparison post-hoc test for comparing different groups. A value of *P* < 0.05 was considered as statistically significant.

## SUPPLEMENTARY MATERIALS TABLES



## Abbreviations

QKI: Quaking
miRs: MicroRNAs
ESCs: Embryonic stem cells
SMC: smooth muscle cell
VSMCs: Vascular smooth muscle cells
SMA: α smooth muscle actin
SMMHC: Smooth Muscle Myosin Heavy Chain
SRF: Serum Response Factor
Myocd: Myocardin
