# Supplementary material for: MicroRNA-214 regulates smooth muscle cell differentiation from stem cells by targeting RNA-binding protein QKI
Source: Oncotarget. 2017 Feb 8;8(12):19866–78. doi: 10.18632/oncotarget.15189 (PMC5386729; doi:10.18632/oncotarget.15189)
Supplement: Supplementary file 1 [file oncotarget-08-19866-s001.pdf]

MicroRNA-214 regulates smooth muscle cell differentiation from stem cells by targeting RNA-binding protein QKI

SUPPLEMENTARY TABLE

Supplementary Table 1: Primer sets used in the present study

Gene names	Forward (5'-3')	Reverse (5'-3')	Application
18s	CCCAGTAAGTGCGGGTCATAA	CCGAGGGCCTCACTAAACC	Real-time RT-PCR
U6 snoRNA	CCATACCACCCTGGAAACGC	TACTAACCGAGCCCGACCCT	Real-time RT-PCR
miR-214 (mu)	AACTGGTGTCGTGGAGTCGGC	GGCCTGGCTGGACAGAGTTG	Real-time RT-PCR
SMαA	TCCTGACGCTGAAGTATCCGAT	GGCCACACGAAGCTCGTTATAG	Real-time RT-PCR
SM22α	GAT ATG GCA GCA GTG CAG AG	AGT TGG CTG TCT GTG AAG TC	Real-time RT-PCR
h1-Calponin	GGT CCT GCC TAC GGC TTG TC	TCG CAA AGA ATG ATC CCG TC	Real-time RT-PCR
SM-myh11	AAG CAG CCA GCA TCA AGG AG	AGC TCT GCC ATG TCC TCC AC	Real-time RT-PCR
SRF	CCTACCAGGTGTCGGAATCTGA	TCTGGATTGTGGAGGTGGTACC	Real-time RT-PCR
Myocd	TCAATGAGAAGATCGCTCTCCG	GTCATCCTCAAAGGCGAATGC	Real-time RT-PCR
MEF2C	AAGCCAAATCTCCTCCCCCTAT	TGATTCACTGATGGCATCGTGT	Real-time RT-PCR
QKI	TACAGACCGCTGTCATGC	TGGTGCCAATGTGTAGGG	Real-time RT-PCR
SMαA-P1	CATAACGAGCTGAGCTGCCTC	CCAAACAAGGAGCAAAGACG	CHIP assay (with CArG region)
SMαA-P2	GATCAGAGCAAGGGGCTATA	CTACTTACCCTGACAGCGAC	CHIP assay (without CArG region)
SM22α-P1	GCAGGTTCCTTTGTCGGGCCA	CTGCTTGGCTCACCACCCCG	CHIP assay (with CArG region)
SM22α-P2	CTTTAAACCCCTCACCCAGC	ATGACTTGCACTTACAAGG	CHIP assay (without CArG region)
SRF-P_F2/R2	CCTGGCTGGCTTGGCACTCAC	ATCTGGCCGGACGGTGTGATA	CHIP assay
SRF-P_F4/R4	CCCTCTTCTGCCCTGCAGTCCT	CCGCGATTCCGTGGGAGGGA	CHIP assay (Adjacent region)
MEF2c-P_F1/R1	AGTCAGGACAGTGGCTTAGCGT	CGGGTTAGTCTCTGACAGTGGACC	CHIP assay
MEF2c-P_F4/R4	GCTCCAGTGTGCGATGTGCCA	TCCGCAGCAAGCCAGTTTCC	CHIP assay (Adjacent region)
Myocd-P_F1/R1	AGGCAGCCTGTTGTAGGCTCG	GAGAGGAGGTGGAGCCCTGCT	CHIP assay (Adjacent region)
Myocd-P_F3/R3	CGGGAGTTGCAAGCCAACCCA	TCCCCAGCTTACTGCAGGGCT	CHIP assay
